# Cardiac rehabilitation adapted to transient ischaemic attack and stroke (CRAFTS): a randomised controlled trial

**DOI:** 10.1186/1471-2377-9-9

**Published:** 2009-02-23

**Authors:** Olive Lennon, Catherine Blake

**Affiliations:** 1 School of Physiotherapy and Performance Science, University College Dublin, Dublin, Ireland

## Abstract

**Background:**

Coronary Heart Disease and Cerebrovascular Disease share many predisposing, modifiable risk factors (hypertension, abnormal blood lipids and lipoproteins, cigarette smoking, physical inactivity, obesity and diabetes mellitus). Lifestyle interventions and pharmacological therapy are recognised as the cornerstones of secondary prevention. Cochrane review has proven the benefits of programmes incorporating exercise and lifestyle counselling in the cardiac disease population. A Cochrane review highlighted as priority, the need to establish feasibility and efficacy of exercise based interventions for Cerebrovascular Disease.

**Methods:**

A single blind randomised controlled trial is proposed to examine a primary care cardiac rehabilitation programme for adults post transient ischemic attack (TIA) and stroke in effecting a positive change in the primary outcome measures of cardiac risk scores derived from Blood Pressure, lipid profile, smoking and diabetic status and lifestyle factors of habitual smoking, exercise and healthy eating participation. Secondary outcomes of interest include health related quality of life as measured by the Hospital Anxiety and Depression Scale, the Stroke Specific Quality of Life scale and WONCA COOP Functional Health Status charts and cardiovascular fitness as measured by a sub-maximal fitness test.

A total of 144 patients, over 18 years of age with confirmed diagnosis of ischaemic stroke or TIA, will be recruited from Dublin community stroke services and two tertiary T.I.A clinics. Exclusion criteria will include oxygen dependence, unstable cardiac conditions, uncontrolled diabetes, major medical conditions, claudication, febrile illness, pregnancy or cognitive impairment. Participants will be block-statified, randomly allocated to one of two groups using a pre-prepared computer generated randomisation schedule. Both groups will receive a two hour education class on risk reduction post stroke. The intervention group will receive a 10 week programme of supervised aerobic exercises (twice weekly) and individually tailored brief intervention lifestyle counselling. Both groups will be tested on week one and week ten of the programme. Follow-up at 1 year will assess longer term benefits. Analysis will test for significant changes in the key variables indicated.

**Discussion:**

Application of the Cardiac Rehabilitation paradigm to patients with ischaemic stroke or TIA has not been explored despite the obvious overlap in aetiology. It is hoped the anticipated improvement in vascular risk factors and fitness resulting from such a programme will enhance health and social gain in this population.

**Trial Registration:**

Current Controlled Trials ISCTRN90272638.

## Background

Stroke is the third leading cause of death and disability worldwide. It constitutes a formidable burden of disability for patients, their families, health professionals and the wider community. It combines aspects of both acute and chronic disease, and there is increasing evidence that those affected by stroke can benefit to a very significant extent from organised stroke care throughout the course of the illness. Delivering this care requires a combination of skills that draw on neurosciences, cardiovascular medicine, general medicine, the science of ageing, rehabilitation, vascular surgery, and public health[1].

Both coronary heart disease (CHD) and ischaemic stroke share links to many of the same predisposing, potentially modifiable risk factors (hypertension, abnormal blood lipids and lipoproteins, cigarette smoking, physical inactivity, obesity and diabetes mellitus). This highlights the prominent role lifestyle plays in the origin of cardiovascular disease [2]. Modification of multiple risk factors through a combination of lifestyle interventions and appropriate pharmacological therapy is now recognised as the cornerstone of initiatives aimed at prevention of recurrent stroke and acute cardiac events in stroke survivors [3-5].

Falling CHD and stroke mortality rates means a higher prevalence of those at risk of further events thus increasing the need for disease management, secondary prevention and cardiac rehabilitation [6]. Patients with a recent ischaemic stroke or transient ischemic attack (TIA) are at high risk for further vascular events and vascular death [7,8]. At least 15% of stroke survivors will have a second stroke during the next five years, quarter of which prove fatal within four weeks [9]. Prospective studies following TIA found incidence of stroke to be 11% within 90 days. Longer term studies found that while the risk of major vascular events- stroke, myocardial infarct (MI) and vascular death is mainly concentrated in the weeks following TIA, it remains elevated for many years afterwards [10,11].

Current evidence for secondary prevention is dominated by pharmacological interventions, while the evidence available for life-style modifications such as dietary change and physical exercise is less robust. Modification of major cardiovascular risk factors (blood cholesterol, high blood pressure and smoking) is reported to be cost-effective but needs to be better targeted if potential health gain is to be realised [12].

Given the proven benefits of regular physical activity on cardiovascular disease risk factors in able-bodied individuals [13,14] and in the post MI and coronary grafting (CABG) populations [15,16], the support for cardiovascular training in stroke survivors is growing. The need for research into the effects of such programmes has recently been highlighted as a priority by the American Stroke Association (ASA) [2] and Cochrane Review [17].

While it is reported that cardiac disease occurs in up to 75% of stroke survivors, exertion at the levels used in rehabilitation programmes after stroke is associated with low risk of serious cardiovascular complications [2]. The proposed research, which also aims to exercise this population at light to moderate i.e. submaximal intensities of ≤ 60% of their maximal heart rate, poses very low risk to the participants of adverse events and is within the recommended guidelines of the ASA at 50–80%. This is further supported by a study reporting a low frequency of medical complications during inpatient rehabilitation post stroke (No myocardial infarctions) [18]. In a scientific statement on physical activity after stroke the American Heart Association (AHA) espouse the use of aerobic conditioning post stroke to improve cardiovascular risk factors and recommend its use for stroke survivors as part of a comprehensive stroke and cardiovascular risk reduction programme [2].

The concept of cardiovascular fitness for stroke survivors has been addressed in a number of studies to date. The Cochrane review on Physical Fitness Training in Stroke Patients [17] includes 11 clinical trials. Of these however only two [19,20] included measures of aerobic fitness (VO2) and maximal work rate (Watts) as defined outcome variables. Similarly only 2 studies [19,21] described the intensity and progression of fitness training as a percentage of the participants maximal capacity, thus meeting the American College of Sports Medicine (ACSM) guidelines for developing cardiorespiratory fitness [22]. The randomised controlled trial by Potempa et al. [19] found that stroke patients could increase their cardiovascular fitness by a magnitude similar to that of healthy older adults who engage in endurance training. However, as only 7% of patients discharged from rehabilitation post stroke meet the criteria for community walking (e.g. a speed that would allow them to cross a road safely), few stroke patients are able to avail of cardiovascular training through the traditional method of treadmill training used in this study.

A cycle ergometer with adaptive devices as necessary can allow aerobic fitness training in this population of more dependent stroke survivors. Treadmill, bicycle, upper limb ergometry and step training can be used in the TIA and less disabled stroke subjects. Traditionally cycle ergometry studies have looked at exercise capacity and self concept in stroke patients following exercise training only. A study addressing exercise in men with Coronary Artery Disease (CAD) and physical disability [23] demonstrated significant increases in peak left ventricular ejection fraction and high-density lipoprotein cholesterol and decreases in resting heart rate following training.

More recent studies in the stroke population have demonstrated water based exercise [24], treadmill training [25] and a community-based exercise and mobility programme [26] can increase cardiovascular fitness after stroke, but a search of the literature has found no studies exploring the effects of an intensive programme consisting of progressive cardiorespiratory training which conforms to ACSM guidelines, accompanied by lifestyle counselling and targeted smoking cessation. These interventions are common components of 'Comprehensive Cardiac Rehabilitation Programmes' but this paradigm has not been applied to the Stroke and TIA population

### Background work

A pilot study was conducted in 2005 looking at the feasibility of a 10 week cardiovascular training programme in the non-acute ischaemic stroke population [27]. This work highlighted the efficacy of the programme in reducing cardiac risk scores and improving cardiovascular fitness in the stroke population. It also demonstrated the safety of the programme with no adverse events occurring during the exercise regime.

Focus groups were subsequently conducted with 4 groups of community dwelling stroke patients (5–6 participants per group) to establish perceived barriers to healthy lifestyle participation, preferred options for receiving information and advice and to gain end-user input into the final programme development for this clinical trial. Conversations were transcribed verbatim and subjected to thematic coding and analysis as guided by Miles and Huberman [28].

### Reason for expansion of the study

Given the proven safety record and notable benefits, it was decided to expand the study to include the post TIA population. As this population have no acquired physical disability, secondary prevention will have greater cost benefit to the health service by reducing the shared risk factors for the occurrence of stroke (currently estimated at 15% within 5 years) and development of other cardiovascular disease (CVD).

Cochrane Reviews of dietary advice, smoking cessation and stress management counselling as part of a comprehensive cardiac rehabilitation programme (CCR) in the CVD population concluded that:

• Dietary advice appears to be effective in bringing about modest beneficial changes in diet and cardiovascular risk factors over approximately 9 months but longer term effects are not known [29].

• Behavioural counselling for smoking cessation indicated that individual counselling was more effective than control. The odds ratio for successful smoking cessation was 1.56 (95% confidence interval 1.32 to 1.84). A greater effect was not detected by intensive counselling compared to brief counselling (odds ratio 0.98, 95% confidence interval 0.61 to 1.56) [30].

• Quitting smoking is associated with a substantial reduction in risk of all-cause mortality among patients with CHD. The pooled crude RR was 0.64 (95% CI 0.58 to 0.71). This 36% risk reduction appears substantial compared with other secondary preventive therapies such as cholesterol lowering which have received greater attention in recent years [31].

• Overall psychological interventions showed no evidence of effect on total or cardiac mortality, but did show small reductions in anxiety and depression in patients with CHD. Similar results were seen for stress management interventions when considered separately [32].

Although brief clinician interventions addressing physical activity and diet have been less studied than smoking cessation programmes, evidence exists that these types of interventions can influence patients to consider and adopt changes in how they eat and their physical activity levels [33,34]. Success of programmes to increase walking and other types of physical activity [34], change dietary behaviour, such as encouraging people to eat more fruit and vegetables in the 5-a-day program [36] further support clinicians' efforts to intervene in these areas.

Based on the evidence of effect of these interventions as part of CCR, adoption of the paradigm to the Stroke and TIA population appears to be warranted.

The aims of the proposed study are to determine whether a 10 week comprehensive cardiac rehabilitation programme for adults post TIA and ischaemic stroke including aerobic training, dietary and exercise advice, life skills and smoking cessation counselling:

(i) Reduces overall cardiac risk score, calculated from Blood Pressure, lipid profile, age, sex and diabetic status [38].

(ii) Increases participants' adherence to the Fourth Joint European Societies' Task Force on Cardiovascular Disease Prevention in Clinical Practice for a high risk group [39] eg by effecting a change in smoking habit as demonstrated by % reduction in smoking, healthy heart diet compliance by daily fruit(2) and veg (3)intake, habitual exercise habit, i.e. % taking moderate and/or vigorous exercise 3 times per week extrapolated from the International Physical Activity Questionnaire (IPAQ) [40].

Secondary outcomes of interest associated with the programme include: (i) Cardiovascular fitness as calculated by a submaximal cycle ergometry test of VO2 max and peak rate pressure product (PRPP). (ii) Health Related Quality of Life as measured by the Hospital Anxiety and Depression Scale scores [41], Stroke Specific Quality of Life Index [42] and Functional Health Status Charts COOP/WONCA [43].

## Methods

The Cochrane criteria for assessing methodological quality of clinical trials and the Consort checklist for publication were consulted to ensure methodological rigor in the design of this study.

### Randomisation procedure

Since blinding to the cardiac rehabilitation programme intervention is not possible, a randomised, controlled, single-blind trial is proposed. Patients recruited for this trial will be randomly assigned to a cardiac rehabilitation group and a non-cardiac rehabilitation group. Participants will be stratified into Stroke and TIA groups and block randomisation using blocks of 12 will be performed using a computer generated randomisation schedule (SPSS v12.0) prepared by an independent party in advance. Concealed allocation will be ensured through the use of opaque sealed envelopes.

### Observer blinding

All measurements (baseline, post intervention and 1 year review) will be made by an independent assessor (CB) blind to the treatment allocation.

### Intervention

The cardiac rehabilitation group will participate in a 10 week programme including 8 weeks physiotherapy led (OL), individually tailored aerobic exercise for one hour twice weekly at up to 60% of their maximal heart rate. They will receive a two hour didactic lecture addressing modifiable risk factors for stroke and stress management (OL and AC). In addition individual brief smoking cessation counselling, exercise and dietary advice (OL) including information leaflets in accordance with the WHO guidelines for secondary prevention in low and medium resource settings [44] will be provided to those failing to meet the recommended guidelines.

To minimise the Hawthorne effect, the control group will attend the two hour risk reduction lecture as outlined above.

### Participant Recruitment

Subjects with ischaemic stroke will be recruited from the outpatient Stroke Rehabilitation Unit, Baggot Street Community Hospital, from the 4 community based stroke support groups. TIA subjects will be recruited from a TIA clinic in a tertiary hospital in the greater Dublin. Information leaflets will be distributed to potential subjects fulfilling the inclusion criteria in the Stroke Rehabilitation Unit database, Baggot Street Community Hospital and TIA clinic in AMNCH Hospital, with the consent of the Consultant Neurologist in this centre. Interested subjects will then contact the principle researcher (OL) should they wish to participate. Talks will be given at each support group branch meeting and information leaflets distributed. Participants will then be screened by phone to ensure they fulfil the inclusion criteria and that none of the exclusion criteria apply. The American College of Sports Medicine PARQ [22] questionnaire will be incorporated in the screening process to exclude subjects unsuitable for exercise testing. The General Practitioners of those attending the support groups will be contacted to give permission for patients to attend the programme and to consent to access medical records where necessary. A one week period between the information dissemination and consent will be given to allow due time for participants to consider the study.

### Inclusion Criteria

Medically stable post ichaemic stroke or (> 3 months) post TIA, referred from specialist tertiary centres. > 1 year post ischaemic stroke with G.P. consent from community services. Over 18 years of age of either sex.

### Exclusion Criteria

O2 dependence, angina, unstable cardiac conditions, unstable diabetes, major medical conditions, claudication, febrile illness, pregnancy or cognitive impairment.

**Sample size calculations **based on the ability to detect significant differences in the key outcome variables with 80% power and p < 0.05 are indicated in table 1. Cardiac risk data was taken from the pilot study and adherence to European guidelines for lifestyle participation in a high risk CVD group was taken from baseline data of the first 50 subjects recruited to the CRAFTS trial. The formulae for detecting change in % was n > = K [P_1_(1 - P_1_) + P_2 _(1-P_2_)]/Δ^2^. The formula for detecting a change in means was n >= 2Kσ^2^/Δ^2^K = 7.8. Our pilot study [27] resulted in a withdrawal rate of 5% overall. Based on primary outcome measures of adherence to all three European lifestyle guidelines and cardiac risk score reduction with an anticipated drop-out rate of 10%, 144 participants will be recruited. 72 randomised to the intervention group and 72 to the control group.

**Table 1:** Sample size calculations

	**% adherence to European Guidelines on CVD prevention in high risk group(CRAFTS baseline data)**		**Target change to detect %**	**Sample size per group**
Moderate or vigorous exercise for 20 mins 3 or more times/week	17		Increase by 20%	73
Dietary intake of 3 vegetables portions & 2 fruit portions or more/day	21		Increase by 20%	80
No smoking % in stroke and TIA patient population	85		Increase by 15%	61
% subjects fulfilling all 3 criterion	9		Increase by 20%	56
	**Mean (sd) of baseline measures from pilot study in stroke patients**	**Mean change in intervention group – pilot study**	**Target change to detect**	
Cardiac Risk Score	9.4 (6.80)	1.52	4.0	72
VO2 exercise test	10.87 (1.71)	1.48	1.0	46
HAD – Anxiety subscale	6.13 (3.71)	0.86	1.5	54

### Consent

Subjects will sign an informed consent statement approved by AMNCH and University College Dublin Ethics Committees prior to participation. Consent will also be sought to access medical records in the hospital of admission and also to contact their General Practitioners (GP). The GP's of all consenting participants will be informed of their agreement to participate and will be asked to inform the study team should they know of any contra-indications to exercise. A further medical screening will be provided prior to sub-maximal exercise testing.

### Outcome Measures

The outcomes of interest can be categorised under the following headings:

1. Change in cardiovascular disease risk

2. Change in health behaviour

3. Change in body objective physical status and physical fitness indices

4. Change in self-reported functioning

The variables and measurement instruments which will be employed are summarised in table 2. Measures will be taken at baseline, at completion of the 10 week intervention programme and at 1 year post intervention. Both the short-term and long-term outcomes are illustrated.

**Table 2:** Variables and measurement instruments

	Instrument	Baseline	Post Intervention	1 year Follow up
**Cardiac Risk**				
Cardiac risk score Waist girth	(Wilson et al 1998) [38] Tape measure	√ √	√ √	√ √
Waist girth	Tape measure	√	√	√
Lipid profile (Cholesterol, trigylceride, HDL, LDL)	Biochemical analysis	√	√	√
Blood Pressure	Sphygomanometer	√	√	√
Mortality				√
Non-fatal CVD/CeVD Event				√
**Physical Fitness & Function**				
Resting HR	Polar heart rate monitor	√	√	
Height, Weight, BMI	Weighing Scales & Meter Rule	√	√	√
Aerobic Fitness – VO2	Astrand Rhyming submaximal exercise test with cycle ergometer	√	√	√
Peak Rate Pressure Product	Polar heart rate monitor and Sphygomanometer	√	√	√
**Self-Reported Function**				
Physical activity and exercise participation % taking moderate and vigorous exercise 3 times per week	International Physical Activity Questionnaire(IPAQ) [40].	√	√	√
Health Related Quality of Life	COOP/WONCA Functional health status charts [43] Stroke Specific Quality of Life Index [42]	√ √	√ √	√ √
**Health Behaviours**				
Smoking – % smoking Diet	Self Reported Status Fruit & vegetable servings/day	√ √	√ √	√ √

### (a) Treatment group

The Intensive Rehabilitation Programme will consist of a 10 week schedule. Baseline measures will be taken during week 1, and an individually tailored exercise training programme prescribed. Training intensity will conform to the recommendations of the American Heart Association, American Stroke Association and American College of Sports Medicine guidelines for aerobic training in patients with cardiac risk [22]. During the subsequent 8 weeks the participants will attend twice weekly for one hour individually tailored fitness training (warm up, cycle ergometry (Motomed or Tunturi cycle ergometer) using either the upper or lower limbs, treadmill training, step work at up to 60% of their maximal heart rate). They will also attend the two hour risk reduction lecture and receive smoking cessation, physical activity and diet counselling appropriate to their needs, in accordance with the World Health Organisation (WHO) guidelines [44]. Reassessment will take place on the 10^th ^week.

The exercise sessions will be supervised by a physiotherapist (OL). Exercise will be stopped if any of the following adverse events occur: chest pain, dizziness, malaise, heart rate in excess of 60% maximal heart rate, oxygen saturation levels lower than 90% and request from participant to stop in accordance with American Heart Association guidelines for exercise testing [45]. Staff with cardiac resuscitation and defibrillation training will be on site during the exercise sessions and the cardiac arrest procedures of the community hospital will apply, immediate first aid and emergency services will be called should an adverse cardiac event occur.

### (b) Control Group

Subjects allocated to the control group will have baseline assessment on week 1 and re-assessment on week 10. They will attend the two hour didactic lecture on risk reduction post stroke and stress management.

### Data Collection and Statistical Analysis

Data collected will be recorded on a pro-forma collection sheet and will be entered onto Microsoft Excel and the Statistical Package for the Social Sciences (SPSS v12.0). Analysis will be conducted on an intention to treat basis and withdrawals from the programme will be followed up to ensure complete data. A last measurement carried forward method will be used to predict outcome where drop-outs occur [46]. Baseline data will be reported using descriptive and frequency statistics.

Change scores occurring over the 10 week trial period and between baseline and 1 year will be compared between intervention and control groups using:

▪ Independent t tests and one-way ANOVA where data are interval or ratio level and conform to the assumptions of normality i.e. Cardiac Risk Score, VO2, Maximal work output (Watts), Peak Pressure Rate Product, Lipid profile (Total Cholesterol, HDL, LDL, Triglyceride).

▪ Non-parametric tests (Mann Whitney and Kruskall Wallis tests) will be used for ordinal level data (Hospital Anxiety and Depression Scale, Stroke Specific QoL) or where a normal data distribution is not found.

▪ Categorical variables will be analysed using Chi square tests (% smoking, % taking moderate and vigorous exercise 3 times per week, % meeting 2 fruit/3 veg daily guidelines).

### Ethical Considerations and Confidentiality

The study conforms to the guidelines of University College Dublin Human Research Ethics Sub-committee (Ref HREC-19-06-Blake). Ethical approval has also been obtained from Saint James's Hospital SHJ/Adelaide, Meath and National Children's Hospitals AMNCH Research Ethics Committee (Ref REC: 2008/07/03)

All participants will be volunteers. Informed consent will be sought from participants following the distribution of an information leaflet and screening for exclusion criteria. Participants will be free to withdraw without prejudice at any time. Since the type of cardiac rehabilitation programme under investigation is not currently available to the Stroke/TIA population, there are no ethical considerations relating to the allocation of participants to a control group.

All information collected relating to participants will be confidential and will be stored securely in a locked cabinet in University College Dublin. Participants will be given a code on entering the study. This will be the only link between the participants name and data.

## Discussion

This study builds on a promising pilot study resulting in a reduced cardiac risk score and improved fitness profiles and sense of well being. It is hoped that success of the Cardiac Rehabilitation Paradigm adapted to the Stroke and TIA population will help inform the care-pathway and enhance health and social gain in this population. While a reduction of morbidity and mortality rates are not stated outcomes of this study, the incidence of new cardiovascular disease events (Cardiac death, non fatal MI, CABG, PTCA, angina, stroke death, non-fatal stroke and TIA) will be monitored in the intervention and control groups over the 1 year follow up period. It is acknowledged that the study is not powered to determine differences in morbidity and mortality, but it is planned to build on this study to determine longer term benefit.

## Competing interests

The authors declare that they have no competing interests.

## Authors' contributions

Both authors have contributed to the development and writing of the study protocol and in the drafting of this manuscript.

## Pre-publication history

The pre-publication history for this paper can be accessed here:


